# GlycoRNA-related cellular program scoring identifies Siglec- and myeloid-associated tumor microenvironments for glycoRNA validation

**DOI:** 10.3389/fimmu.2026.1928501

**Published:** 2026-10-13

**Authors:** Mowei Kong, Xiangfeng Guan, Yuling Jing, Zheng Xie, Xingwei Zhao, Shengyu Huang, Yang Yu, Chunxiang Zhang

**Affiliations:** 1 Department of Cardiology, The Affiliated Hospital of Southwest Medical University, Luzhou, Sichuan, China; 2 Department of Orthopedics, Zigong Fourth People’s Hospital, Zigong, Sichuan, China; 3 Key Laboratory of Medical Electrophysiology, Ministry of Education, Institute of Cardiovascular Research, Luzhou, Sichuan, China; 4 Department of Anesthesiology, The Affiliated Hospital of Southwest Medical University, Luzhou, Sichuan, China; 5 Basic Medicine Research Innovation Center for Cardiometabolic Diseases, Ministry of Education, Southwest Medical University, Luzhou, Sichuan, China; 6 Nucleic Acid Medicine of Luzhou Key Laboratory, Luzhou, Sichuan, China

**Keywords:** expression-based scoring, glycoRNA-potential, myeloid cells, Siglec, tumor microenvironment

## Abstract

**Background:**

Glycosylated RNAs (glycoRNAs) represent a recently recognized connection between RNA biology and cell-surface glycobiology. However, whether glycoRNA-related processes are relevant to human tumors remains unclear, partly because direct glycoRNA measurements are not yet available across large cancer cohorts. We therefore used transcriptomic data to identify tumor settings in which glycoRNA-related programs may be most suitable for further experimental validation.

**Methods:**

We curated a glycoRNA-focused literature evidence base and manually reviewed the top extracted relations to define gene modules related to RNA identity, N-glycan and sialylation pathways, RNA trafficking, and lectin/Siglec recognition. The glycoRNA-related cellular program score is an expression-based composite score representing coordinated glycoRNA-related cellular programs, including RNA processing, glycan biosynthesis, trafficking, and lectin/Siglec recognition. It is designed to prioritize tumor contexts for experimental glycoRNA validation. The score was applied to 9,186 TCGA tumors from 32 cancer types and analyzed together with clinical variables, leukocyte fraction, immune signatures, Siglec/checkpoint/myeloid-axis scores, and non-overlapping sensitivity models. Glioblastoma single-cell and spatial transcriptomic datasets were used for additional context-level validation.

**Results:**

Higher glycoRNA-related cellular program scores were observed in several tumor types, including stomach adenocarcinoma, glioblastoma, lung adenocarcinoma, esophageal carcinoma, mesothelioma, pancreatic adenocarcinoma, breast cancer, and lung squamous carcinoma. Survival analyses did not show robust FDR-significant associations after adjustment for age, sex, stage, and tumor type, suggesting that the score should not be interpreted as a general prognostic biomarker. In sensitivity analyses that removed immune and Siglec-related genes from the predictor, the score remained associated with Siglec-decoder, myeloid, checkpoint, and integrated niche features after adjustment for leukocyte fraction and cancer-type effects. Within-cancer meta-analysis supported these associations across cancer types, although effect sizes varied. In glioblastoma, higher scores were found in tumor, myeloid, mesenchymal, and niche-associated compartments in single-cell and spatial analyses.

**Conclusions:**

This study presents an expression-based map of glycoRNA-related cellular program across human cancers. The results suggest that glycoRNA-related transcriptional programs tend to occur in immune- and myeloid-enriched tumor microenvironments, particularly those involving Siglec-associated features. These findings prioritize tumor contexts for biochemical, imaging, spatial, and perturbation-based glycoRNA validation.

## Introduction

Cell-surface glycans regulate tumor immune recognition, receptor signaling, trafficking, invasion, and therapeutic response (1). In parallel, extracellular and cell-surface RNAs have been linked to intercellular communication, innate immune sensing, and vesicle-associated signaling. The discovery that RNAs can be glycosylated and displayed at the cell surface suggests a biochemical bridge between RNA biology and glycobiology (2, 3). However, the tumor microenvironments in which glycoRNA-related mechanisms may operate, and the immune contexts associated with these regulatory processes, remain incompletely defined.

Although recent methodological advances enable targeted detection of glycosylated RNA or RNA-associated glycan chains under specific experimental conditions, direct high-throughput glycoRNA profiling across large tumor cohorts is not yet feasible (4). Consequently, computational mapping approaches cannot quantify glycoRNA abundance or directly infer function, but they can identify contexts in which RNA-processing factors, glycan-biosynthetic enzymes, intracellular transport machinery, and glycan-decoding effectors are coordinately expressed (5). Such context-specific ranking may improve the efficiency of follow-up experiments, particularly because current glycoRNA detection techniques remain specialized, low-throughput, and not yet implemented in routine clinical or large-scale cohort studies.

This study develops a constrained transcriptomic score to estimate the potential for glycoRNA-related biology from bulk and single-cell expression data. The score integrates expression patterns from four related gene modules: RNA processing, glycan biosynthesis, vesicular or plasma-membrane trafficking, and lectin/Siglec-mediated molecular recognition. We applied this metric to two complementary datasets: the TCGA pan-cancer cohort and a single-cell RNA-seq dataset from glioblastoma. The score was then examined together with clinical annotations, computational estimates of immune infiltration, and the expression of immune checkpoint and Siglec-axis genes, allowing the results to be interpreted in a broader biological context. It is important to emphasize that this metric is inferential. The score does not represent direct detection of glycosylated RNA molecules, but rather captures transcriptional signals that may indicate glycoRNA-related functional potential. Using this metric, we identified tumor types and glioblastoma cell states with elevated glycoRNA-related cellular program scores. These higher scores were associated with immune-enriched microenvironments, increased Siglec expression, and upregulation of immune checkpoint pathways, but these expression-based relationships should not be interpreted as evidence that glycoRNA drives immune infiltration, binds Siglecs in tumors, or predicts immunotherapy response.

## Methods

### Literature collection, extraction, and relation audit

RNA glycosylation and glycoRNA-related literature records were collected from metadata, abstracts, and available full-text resources using glycoRNA-centered search terms, including glycosylated RNA, glycoRNA, RNA glycosylation, cell-surface RNA, extracellular RNA, sialylated RNA, N-glycan RNA, Siglec glycoRNA, and RNA-associated glycan. Records were screened for relevance to glycoRNA detection, RNA-associated glycans, glycan biogenesis, surface or extracellular-vesicle localization, lectin/Siglec recognition, cancer, or immune-cell contexts. Articles focused only on general RNA modification or general glycosylation without a plausible RNA-glycan connection were excluded from the core evidence set. Entity and relation extraction was used to identify RNA species, glycan and glycosylation pathways, surface or extracellular-vesicle contexts, lectin/Siglec decoders, tumor types, immune cell states, and experimental methods. Extracted relations were aggregated into a knowledge graph. The top 100 relations, ranked by recurrence, entity normalization confidence, and proximity to glycoRNA-specific evidence snippets, were audited row by row against extracted evidence snippets from a glycoRNA-focused literature corpus (2, 3, 6). Audit categories were retain_core_text_supported, retain_contextual_support, revise_entity_or_scope, and exclude_from_core_claims. The final evidence table reports PMID/DOI, extracted entities, relation type, evidence sentence, evidence-confidence category, and whether the relation was used for score construction. This audit was designed to support transparent module definition rather than to establish a complete systematic review.

### GlycoRNA-related cellular program seed modules

The glycoRNA-related cellular program score is an expression-based composite score representing coordinated glycoRNA-related cellular programs, including RNA processing, glycan biosynthesis, trafficking, and lectin/Siglec recognition. It is designed to prioritize tumor contexts for experimental glycoRNA validation. Seed modules were derived from the curated literature knowledge base and grouped into glycoRNA core literature genes, N-glycan/OST biogenesis, sialylation and nucleotide-sugar metabolism, N-glycan branching, RNA processing and surface trafficking, and lectin/Siglec decoding. Gene symbols were standardized and matched to expression matrices using GENCODE annotation where required. Complete gene lists, source evidence, overlap annotations, and TCGA coverage are provided in **Supplementary Table 4**. The 74 genes were separated into core glycoRNA genes and support-pathway genes to distinguish direct literature evidence from pathway-level contextual support. Core evidence comprises sequence-resolved surface-RNA observations, gene-specific glycoRNA-related perturbation assays, and receptor-specific recognition studies; the supplementary table specifies the assay type, experimental system, and source for each gene. Support-pathway annotations describe established gene functions in RNA processing, glycan biosynthesis, sialylation, trafficking, and lectin recognition. These evidence categories are recorded separately from the six scoring modules, whose gene membership and score calculation were retained. For the supplemental bulk analyses, the supplied sample-level module scores were retained, and the overall score was verified as the arithmetic mean of the six module scores. The lectin/Siglec-module-removed score was the mean of the remaining five modules. Continuous variables were standardized for regression as described below. Knowledge-based gene-set scoring provides the conceptual framework for module aggregation (7).

### TCGA pan-cancer scoring

Alternative weighting sensitivity analysis was performed using the six matched seed modules. The primary alternative used matched-gene-proportion weights (3, 18, 12, 10, 17, and 14 genes; weights normalized to sum to one), whereas the reference score used the arithmetic mean. A second alternative used the signed first principal component after module-wise centering and scaling; its orientation was fixed by positive correlation with the reference score. The analysis included 9,186 records representing 9,100 distinct samples across 32 cancer types. Cancer-type summaries, sample-level correlations with the reference score, and correlations with the Siglec-decoder, myeloid, checkpoint, and integrated niche outcomes are reported in the supplementary sensitivity files. An outcome-trained weighting model was not fitted because the supplied sample-level matrix contained the evaluation outcomes rather than an independent training outcome.

TCGA tumor RNA-seq TPM data from the UCSC Xena Toil recompute resource were filtered to tumor samples using phenotype annotations (8). The recomputed scoring matrix used for reviewer-requested supplemental analyses included 9,186 tumor samples, 32 cohorts, and 74 matched seed genes. Cohort summaries were computed for module-level and overall scores.

One sample per patient per cancer type was retained for primary analyses; additional samples were evaluated in sensitivity analyses using patient-clustered standard errors. Patient IDs were used only to identify repeated samples within cancer type and were not included as model predictors; sample IDs were retained as observation-level identifiers and were not used to encode patient identity. Regression models that included more than one sample per patient used patient-clustered standard errors.

### Survival and clinical modeling

TCGA survival endpoints were integrated with the TCGA Clinical Data Resource (9). Median-split Kaplan-Meier/log-rank screening was performed for cohorts with sufficient samples and events. Cox proportional hazards models used score z-score, age z-score, sex, and stage z-score. The pan-cancer model additionally included tumor-type fixed effects. P values were adjusted using Benjamini-Hochberg FDR. Cox analyses were interpreted as exploratory because several cohorts had incomplete stage annotations, limited events, or convergence warnings.

### Immune association analyses

Leukocyte fraction estimates and PanImmune signature scores were downloaded from TCGA PanImmune resources (10). Spearman correlations were calculated between the glycoRNA-related cellular program score and leukocyte fraction, PanImmune signatures, and expression of checkpoint, Siglec, galectin, and myeloid lectin genes. Axis genes were extracted from the same TCGA expression matrix used for scoring. FDR correction was applied within analysis families. The supplemental analysis assessed each of the six component modules against the total score using Spearman correlation, with Benjamini-Hochberg adjustment across these six tests (**Supplementary Table 25**; **Supplementary Figure 4**). These correlations describe the relationship of the component modules to their composite score.

### GBM single-cell analysis

A glioblastoma h5ad expression matrix was scored using the same seed modules and interpreted in relation to established GBM cellular-state concepts (11). Cell annotations were retained from the original h5ad file. The original scoring matrix contained 123,236 cells across 17 annotation categories. Cells annotated as Doublets were excluded from visualization and Mann-Whitney U tests. Mann-Whitney U tests compared each cell type, anatomical zone, or Neftel class against all remaining cells, followed by FDR correction. Because very large cell numbers can produce highly significant P values for small effects, cell-level tests were interpreted alongside effect sizes, median differences, and patient- or sample-level summaries where annotations were available. The h5ad contained X_tSNE rather than UMAP coordinates; embedding visualizations therefore used tSNE. Independent GBM single-cell or spatial transcriptomic validation is considered necessary before making strong claims about spatially organized glycoRNA-related niches.

### Sensitivity and non-overlapping validation analyses

Composition sensitivity was evaluated using the existing module-removal and score-variant analyses. Each module-removal score was calculated from the remaining components and related to the same immune-axis outcomes. In the supplied sample-level matrix, the immune/Siglec-free, core_no_lectin_siglec, and lectin/Siglec-module-removed variants had identical values. These analyses characterize the contribution of score composition. Covariate-adjusted models separately characterize associations conditional on leukocyte fraction and cancer type.

For adjusted bulk analyses, ordinary least-squares models related each immune-axis outcome to a score variant, leukocyte fraction, and cancer-type fixed effects. Continuous variables were standardized using available values before complete-case filtering. The models included 8,971 score records from 30 represented cancer types. Standardized coefficients, conventional model-based standard errors, and two-sided Student t 95% confidence intervals with 8,939 residual degrees of freedom are reported in **Supplementary Table 25**. The existing FDR values were retained for the 44-model family. All 44 coefficient and standard-error estimates were reproduced from the sample-level matrix. The integrated niche index was the arithmetic mean of the standardized total score, Siglec-decoder score, myeloid score, and checkpoint score; its association is reported as a composite descriptive result. Within-cancer Spearman correlations, random-effects summaries, and label-permutation controls provided complementary sensitivity analyses. The source matrix contained 9,186 score records with 9,100 distinct sample identifiers; the replication retained the original record structure. For **Figure 1**, the immune/Siglec-free (lectin/Siglec-module-removed) score was evaluated within each cancer type against four outcomes. Approximate 95% confidence intervals for individual Spearman correlations were calculated using the Fisher z transformation with standard error 1/sqrt(n - 3). The original Benjamini-Hochberg FDR values were retained from the 384-test family comprising three score variants, four outcomes, and 32 cancer types. Existing random-effects summaries and their 12-test-family FDR values were retained. Descriptive plotting classes were FDR < 0.05 with rho >= 0.30, FDR < 0.05 with 0 < rho < 0.30, FDR >= 0.05, and FDR < 0.05 with rho < 0; the rho = 0.30 threshold was used for visualization.

**Figure 1:**
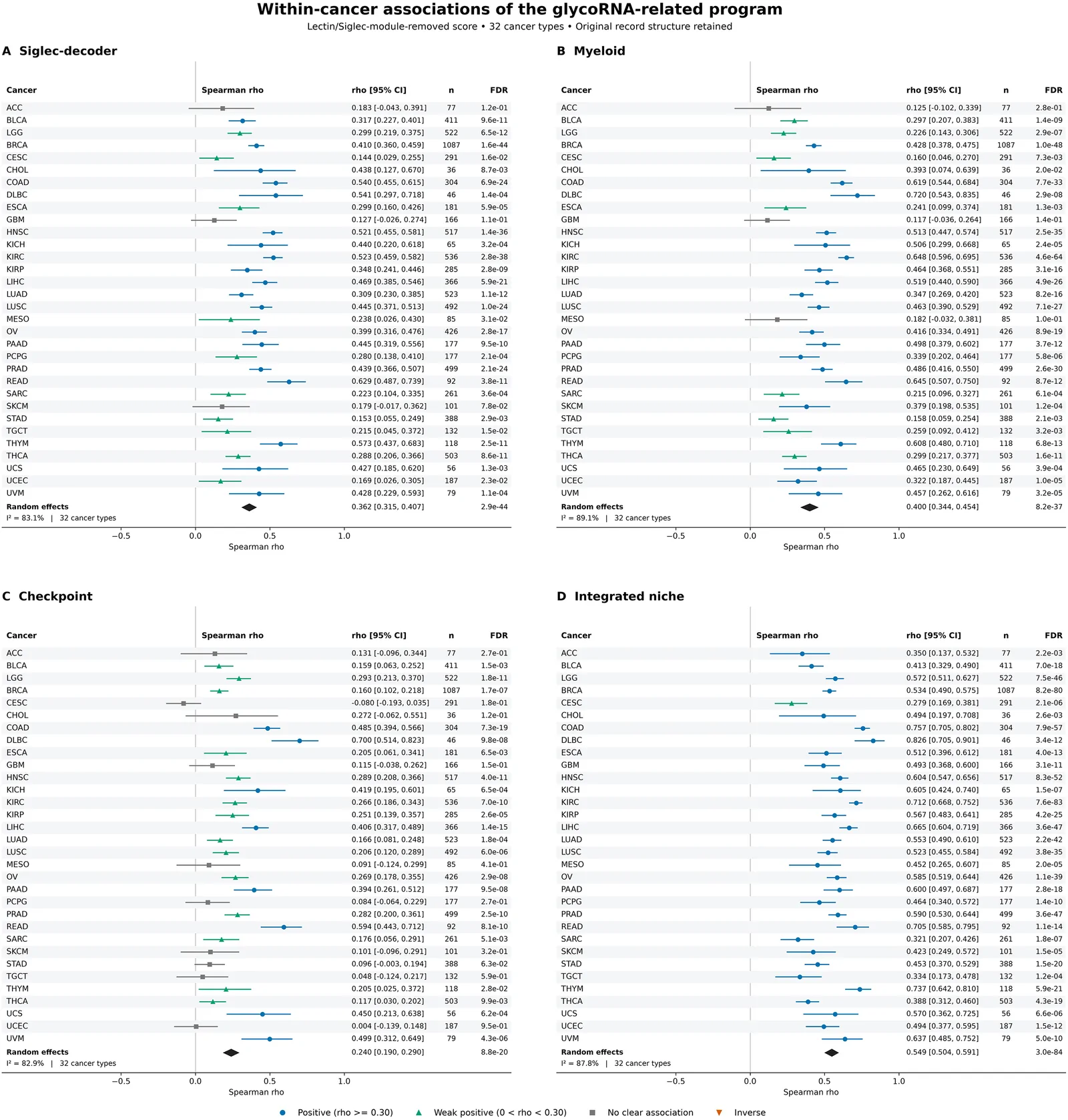
Within-cancer associations between the glycoRNA-related program score and immune/Siglec-axis features. **(A–D)** show associations of the immune/Siglec-free (lectin/Siglec-module-removed) score with Siglec-decoder, myeloid, checkpoint, and integrated niche features, respectively. Each row represents one cancer type and reports Spearman rho, approximate Fisher z 95% confidence interval, record count (n), and the original Benjamini-Hochberg FDR from the 384-test family. Blue circles indicate FDR < 0.05 and rho >= 0.30; green triangles indicate FDR < 0.05 and 0 < rho < 0.30; gray squares indicate FDR >= 0.05; orange inverted triangles indicate FDR < 0.05 and rho < 0. The 0.30 threshold is descriptive. Black diamonds show the existing random-effects summary and its 95% confidence interval; summary FDR values retain the original 12-test family. I2 quantifies between-cancer heterogeneity. Cancer types appear in the same alphabetical order in each panel; full names and TCGA codes are provided in the 128-row source data. The integrated niche index averages the standardized total program, Siglec-decoder, myeloid, and checkpoint scores and is presented as a composite descriptive outcome. Analyses retain the original score-record structure.

### Statistics and visualization

Two-sided nonparametric tests were used unless otherwise stated. Spearman correlation quantified monotonic associations. Benjamini-Hochberg FDR controlled multiple testing. Figures were generated in Python and exported as publication-quality image files.

## Results

### A row-audited glycoRNA evidence base defines the glycoRNA-related cellular program score

We first constructed a glycoRNA-focused evidence base and extracted candidate relations spanning RNA identity, glycan biogenesis, surface or extracellular-vesicle contexts, and lectin/Siglec decoding. The top 100 relations were audited row by row against extracted evidence snippets from a glycoRNA-focused literature corpus (2, 3, 6) and classified by evidence confidence. We then used the curated relations to build a glycoRNA-related cellular program score, while explicitly avoiding claims that the computational score directly measures glycoRNA abundance or function. **Figure 2** summarizes the evidence curation, module construction, TCGA scoring, immune-axis evaluation, and single-cell analyses rather than presenting the knowledge graph as a stand-alone mechanistic discovery.

**Figure 2:**
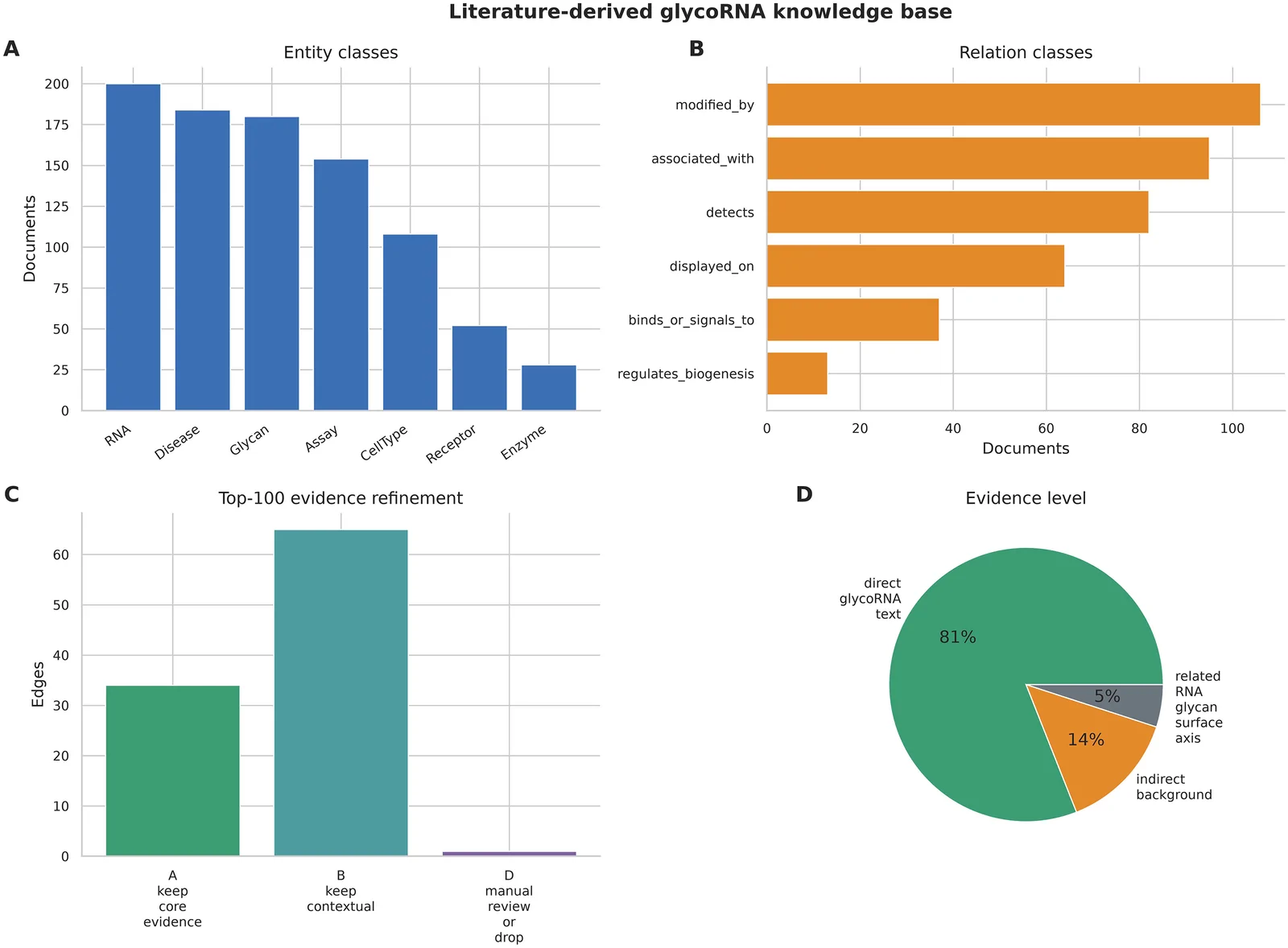
GlycoRNA-related cellular program score construction. **(A)** Entity classes in the glycoRNA/RNA-glycosylation literature corpus. **(B)** Relation classes identified from the literature corpus. **(C)** Row-audited top-100 evidence refinement, showing the retained core evidence, contextual evidence, and manually reviewed or removed relations. **(D)** Evidence levels assigned to the curated relations, including direct glycoRNA evidence, indirect background evidence, and related RNA/glycan surface-axis evidence. The knowledge base is used to construct potential-score modules and does not by itself establish glycoRNA abundance in tumors; the figure emphasizes evidence curation, score construction, and claim boundaries rather than a stand-alone mechanistic knowledge graph. **Figure 3**. Clinical association of the glycoRNA-related cellular program.

### TCGA pan-cancer scoring nominates tumor contexts with elevated glycoRNA-related cellular program programs

The glycoRNA-related cellular program score was recomputed in 9,186 TCGA tumor samples from 32 cohorts, with 74 matched seed genes across six modules. The highest-scoring cohorts included stomach adenocarcinoma, glioblastoma, lung adenocarcinoma, esophageal carcinoma, mesothelioma, pancreatic adenocarcinoma, breast cancer, and lung squamous carcinoma. Module-level heatmaps showed that these rankings were not driven by a single module alone, but by coordinated expression across glycan biogenesis, RNA-processing/trafficking, and lectin/Siglec-decoding components. Reviewer-requested module-ablation and non-overlapping score analyses showed that immune-axis associations persisted after removing lectin/Siglec decoder genes from the predictor. The immune/Siglec-free score remained correlated with Siglec-decoder, myeloid, checkpoint, and integrated niche outcomes (Spearman rho = 0.398, 0.372, 0.248, and 0.541; n = 9,186; FDR <= 8.82e-129), while its correlation with leukocyte fraction was smaller (rho = 0.107; n = 8,971; FDR = 3.17e-24), supporting a signal not fully explained by direct Siglec/checkpoint gene overlap (**Figure 3**).

**Figure 3:**
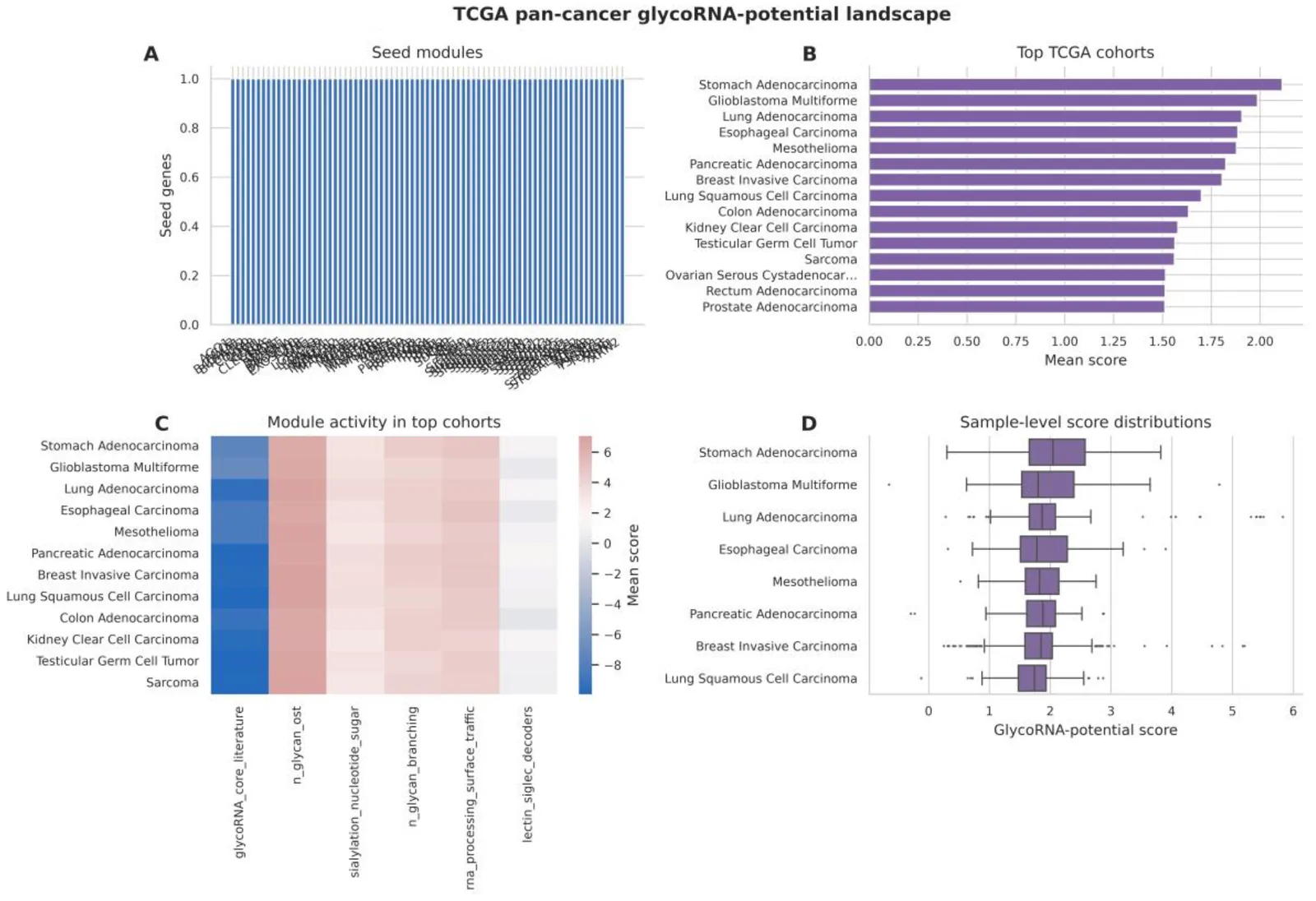
TCGA pan-cancer glycoRNA-related cellular program landscape. **(A)** Seed modules scored across TCGA tumor samples. **(B)** Top TCGA cohorts ranked by mean glycoRNA-related cellular program score. **(C)** Module activity across the highest-scoring cohorts. **(D)** Sample-level distributions of the glycoRNA-related cellular program score in the highest-scoring cohorts. Bar plots show top-scoring cohorts; heatmaps and boxplots show module- and sample-level score distributions. Scores are expression-based context-ranking metrics.

### Clinical association is exploratory and secondary to the transcriptional scoring results

Survival screening identified selected cohort-level associations, but multivariable Cox models adjusting for age, sex, stage, and tumor type produced 19 fitted models and no model passed FDR < 0.1. These results argue against presenting glycoRNA-related cellular program as a universal pan-cancer survival biomarker. Instead, clinical associations are best interpreted as exploratory and secondary to the biological scoring analyses. Survival results are therefore reported as supporting analyses, whereas the main results focus on adjusted non-overlapping immune/Siglec associations and cellular-state mapping (**Figure 4**).

**Figure 4:**
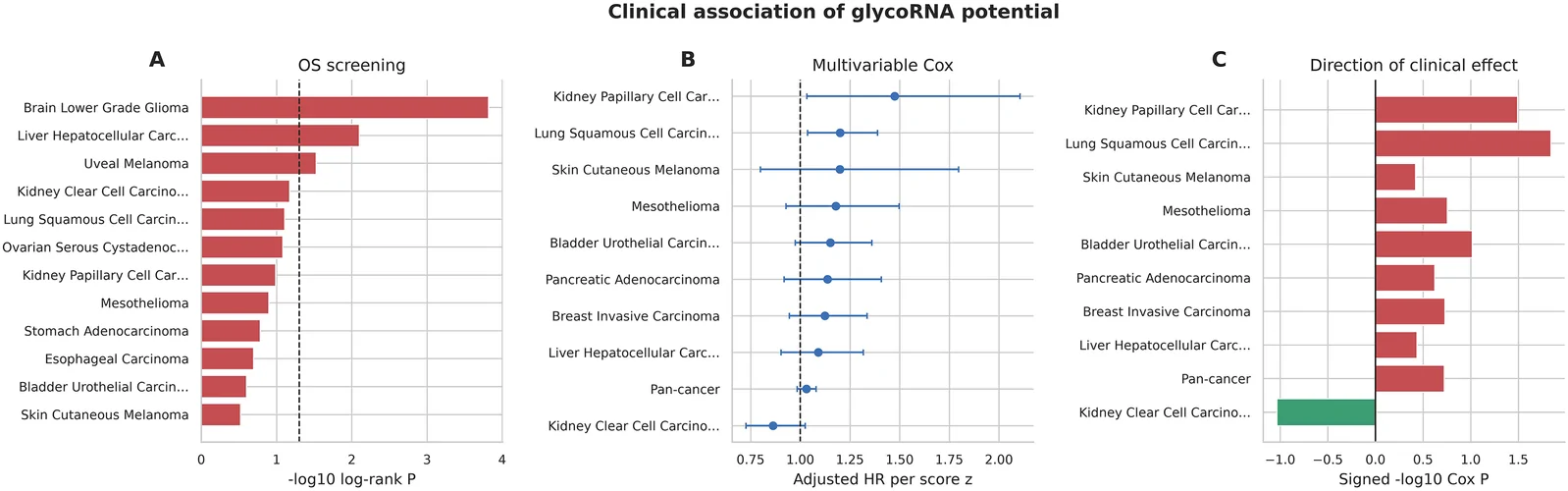
Adjusted non-overlapping immune and Siglec-axis associations. **(A)** Overall-survival screening based on TCGA Clinical Data Resource endpoints. **(B)** Multivariable Cox models adjusted for age, sex, stage, and tumor type. **(C)** Direction of the clinical effect estimated from the multivariable Cox models.

### GBM single-cell analysis maps glycoRNA-related cellular program to tumor and myeloid niches

The macrophage compartments comprised monocyte-derived tumor-associated macrophages (MoTAMs) and microglia-derived tumor-associated macrophages (MgTAMs). Among GBM cells retained after exclusion of Doublets, high glycoRNA-related cellular program scores localized to Tumor (mean = 2.33), Fibroblasts (mean = 1.78), MoTAM (mean = 1.66), MgTAM (mean = 1.34), and VSMCs/Endo (mean = 1.16). Bulk-region cells scored higher than periphery-region cells, and AC/MES-like Neftel states scored higher than OPC/NPC-like states. Independent 10X validation in GSE131928_Neftel most strongly supported co-occurrence with mesenchymal and niche programs (rho = 0.546 and 0.598, respectively; n = 16,201 cells). Direct myeloid, Siglec, and checkpoint correlations in this single-cell validation set were statistically significant but small (rho = 0.065, 0.069, and 0.041), indicating that the independent single-cell signal is driven primarily by malignant-state or niche programs rather than a strong cell-level Siglec/myeloid axis. Spatial GBM validation across five samples provided stronger tissue-context support, with positive spot-level correlations with niche index (rho = 0.524 to 0.706) and mesenchymal score (rho = 0.202 to 0.575), plus sample-dependent but generally positive myeloid, Siglec, and checkpoint associations. High-glycoRNA spots also tended to have neighboring spots with higher niche and mesenchymal scores. These patterns identify cellular and spatial contexts for follow-up rather than definitive glycoRNA localization, and direct biochemical or imaging validation is still required (**Figure 5**).

**Figure 5:**
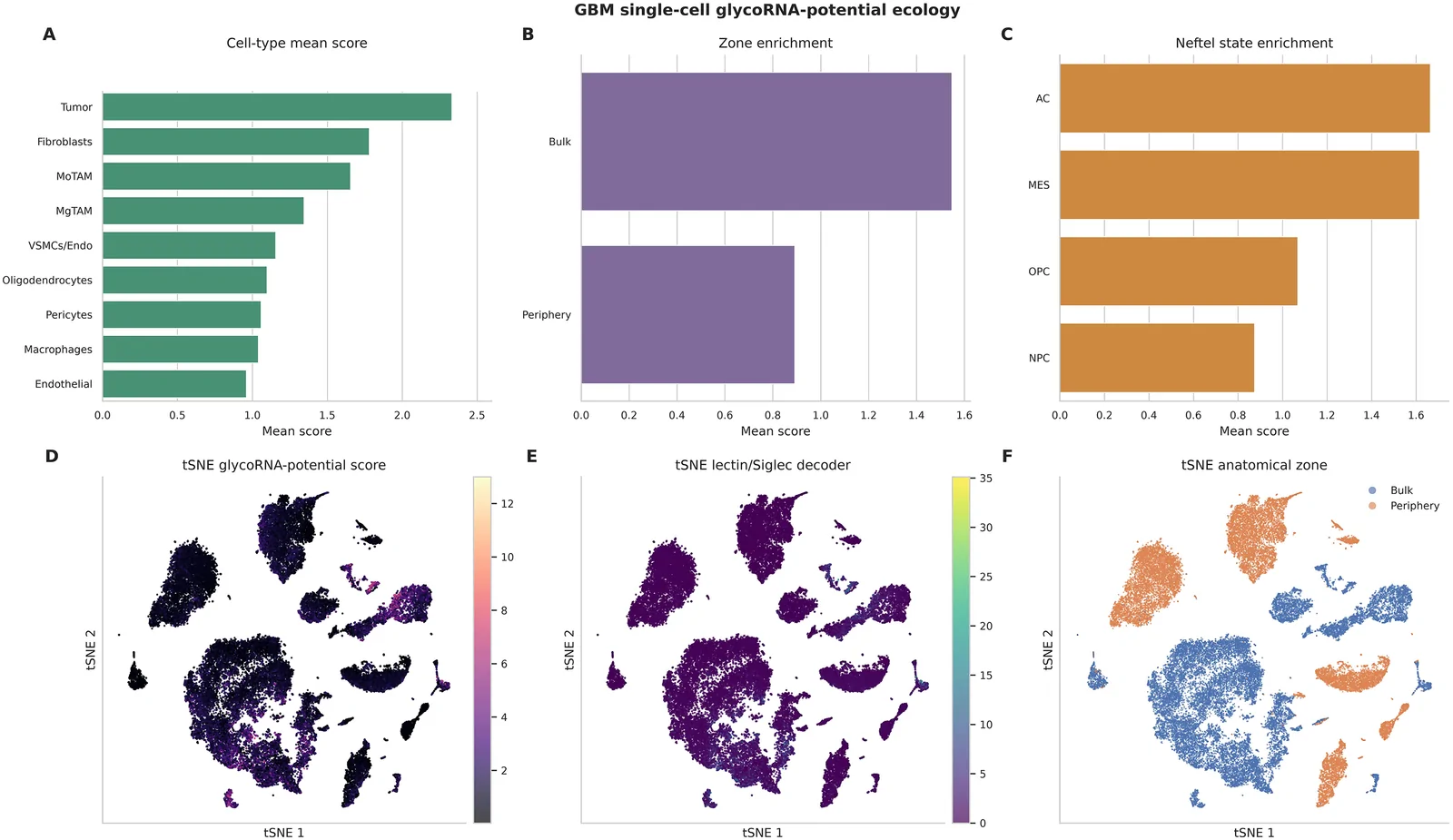
GBM single-cell glycoRNA-related cellular program mapping. **(A)** Mean score across annotated cell types. **(B)** Score enrichment across anatomical zones. **(C)** Enrichment across Neftel cellular states. **(D)** t-SNE embedding colored by the glycoRNA-related cellular program score. **(E)** t-SNE embedding colored by the lectin/Siglec-decoder score. **(F)** t-SNE embedding colored by anatomical zone.

### Integrated expression-based model

By integrating evidence from the literature review, pan-cancer scoring of the glycoRNA-related cellular program based on TCGA data, immune-axis correlation analyses, and single-cell mapping in glioblastoma (GBM), we outline a working model for further investigation. In this model, tumors with higher glycoRNA-related cellular program appear to represent a subset of tumor microenvironments in which RNA-processing programs, glycan biosynthesis, cell-surface or extracellular-vesicle trafficking, and lectin/Siglec-related recognition pathways may be co-expressed.

### Possible mechanistic scenarios and alternative explanations

The present bulk score quantifies tissue-level co-occurrence of the component transcriptional modules and therefore supports two compatible cellular scenarios: a single cell type may express several or all modules, or distinct tumor-microenvironment cell types may contribute different modules, such as tumor cells expressing trafficking genes while infiltrating macrophages express Siglecs. Bulk scoring alone cannot assign each module to a specific cell type or determine whether the modules are expressed by the same cells. Future matched single-cell multi-omic profiling, integrating RNA with protein and glycan measurements, could assign module expression and surface glycoRNA-related features to defined malignant, macrophage, microglial, or other TME populations. Spatial transcriptomics combined with multiplex protein/glycan imaging and cell-segmentation approaches could then test whether module-positive populations occupy the same microanatomical niches, lie in direct proximity, or occur in separate regions. These measurements would distinguish cellular origin from tissue-level co-occurrence and evaluate spatial co-localization of the modules.

This model is hypothesis-generating rather than direct evidence for a causal role of glycoRNA. Its main purpose is to rank tumor contexts and molecular axes for future biochemical validation and functional studies. The present data do not demonstrate that glycoRNA directly regulates immune activity in these tumors (**Figure 6**).

**Figure 6:**
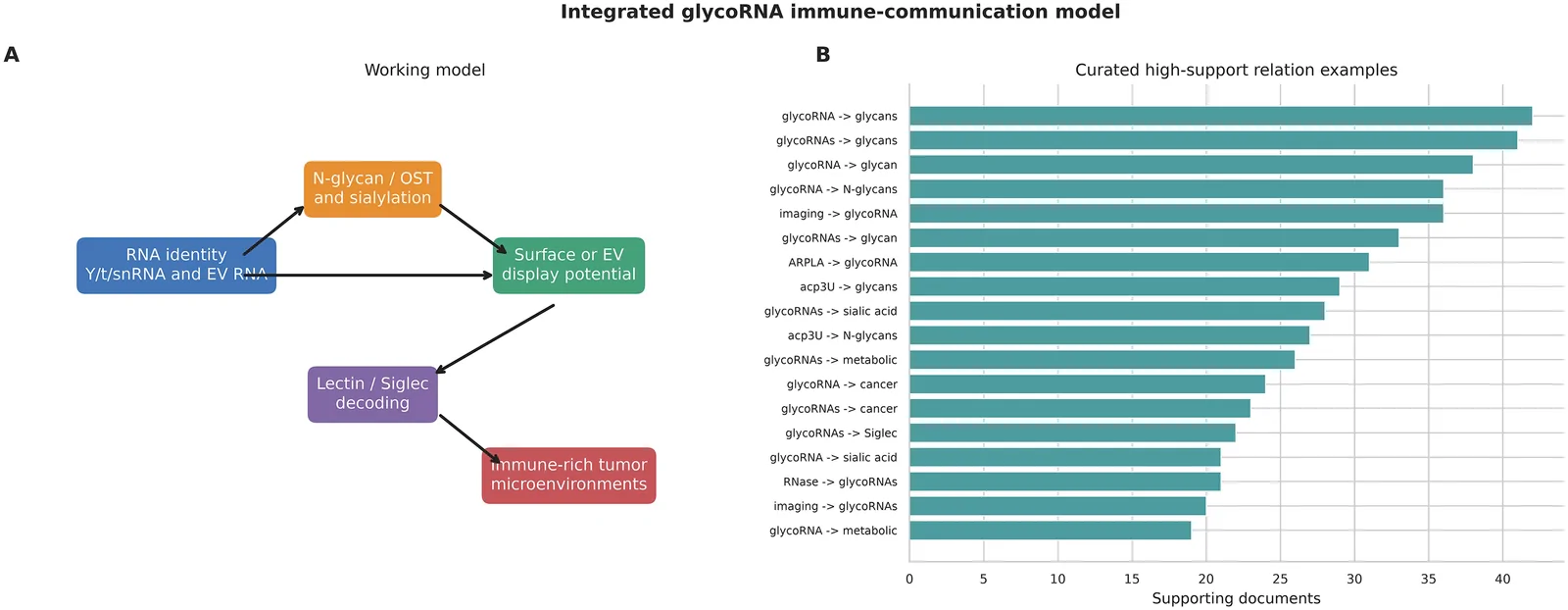
Integrated glycoRNA-related cellular program expression model. **(A)** Leukocyte-fraction associations across cancer types. **(B)** Associations with checkpoint- and Siglec-axis genes.

### GlycoRNA-related cellular program co-occurs with immune infiltration and siglec/checkpoint-axis expression after non-overlapping sensitivity analyses

After adjustment for leukocyte fraction and cancer-type fixed effects, the lectin/Siglec-module-removed cellular program score retained positive associations with Siglec-decoder score (beta = 0.234, 95% CI 0.218–0.251), myeloid score (beta = 0.266, 95% CI 0.249–0.282), checkpoint score (beta = 0.126, 95% CI 0.112–0.140). The corresponding association with the integrated niche index (beta = 0.463, 95% CI 0.449–0.476) is a composite descriptive result because the niche index includes the standardized total score. These models used 8,971 score records from 30 cancer types (**Supplementary Table 25**).

Associations were evaluated both across the pooled cohort and within individual cancer types, with effect sizes varying across tumor contexts. For the immune/Siglec-free score, pooled correlations were rho = 0.362 for Siglec-decoder score (95% CI 0.315 to 0.407; FDR = 2.89e-44), rho = 0.400 for myeloid score (95% CI 0.344 to 0.454; FDR = 8.19e-37), rho = 0.240 for checkpoint score (95% CI 0.190 to 0.290; FDR = 8.85e-20), and rho = 0.549 for the integrated niche index (95% CI 0.504 to 0.591; FDR = 2.98e-84). Heterogeneity ranged from I2 = 82.9% to 89.1%. Within-cancer patterns differed across outcomes: siglec-decoder (29/32 cancer types with positive associations at FDR < 0.05; 19 with rho >= 0.30, 10 with 0 < rho < 0.30, 3 with FDR >= 0.05, and 0 with inverse associations at FDR < 0.05); myeloid (29/32 cancer types with positive associations at FDR < 0.05; 21 with rho >= 0.30, 8 with 0 < rho < 0.30, 3 with FDR >= 0.05, and 0 with inverse associations at FDR < 0.05); checkpoint (22/32 cancer types with positive associations at FDR < 0.05; 8 with rho >= 0.30, 14 with 0 < rho < 0.30, 10 with FDR >= 0.05, and 0 with inverse associations at FDR < 0.05); integrated niche (32/32 cancer types with positive associations at FDR < 0.05; 31 with rho >= 0.30, 1 with 0 < rho < 0.30, 0 with FDR >= 0.05, and 0 with inverse associations at FDR < 0.05). These results describe context-dependent associations across cancer types (**Figure 1**). The integrated niche index is a composite descriptive outcome that includes the standardized total score.

The six component modules correlated positively with the total cellular program score (Spearman rho = 0.409–0.740; n = 9,186; **Supplementary Table 25**; **Supplementary Figure 4**). Removing the lectin/Siglec decoder module changed correlations with the Siglec-decoder, myeloid, and checkpoint outcomes from 0.662, 0.600, and 0.488 to 0.398, 0.372, and 0.248, respectively. In the same covariate-adjusted sample, standardized coefficients for the full and module-removed scores were 0.417 versus 0.234, 0.412 versus 0.266, and 0.238 versus 0.126 for these three outcomes. This attenuation describes the contribution of the lectin/Siglec component to the observed covariation, alongside positive associations of the remaining five-module program. Module removal changes predictor composition, whereas the regression models condition on leukocyte fraction and cancer type. The label-permutation analysis yielded empirical FDR = 1.0 for the four immune-axis outcomes (**Supplementary Table 24**; **Figure 7**).

**Figure 7:**
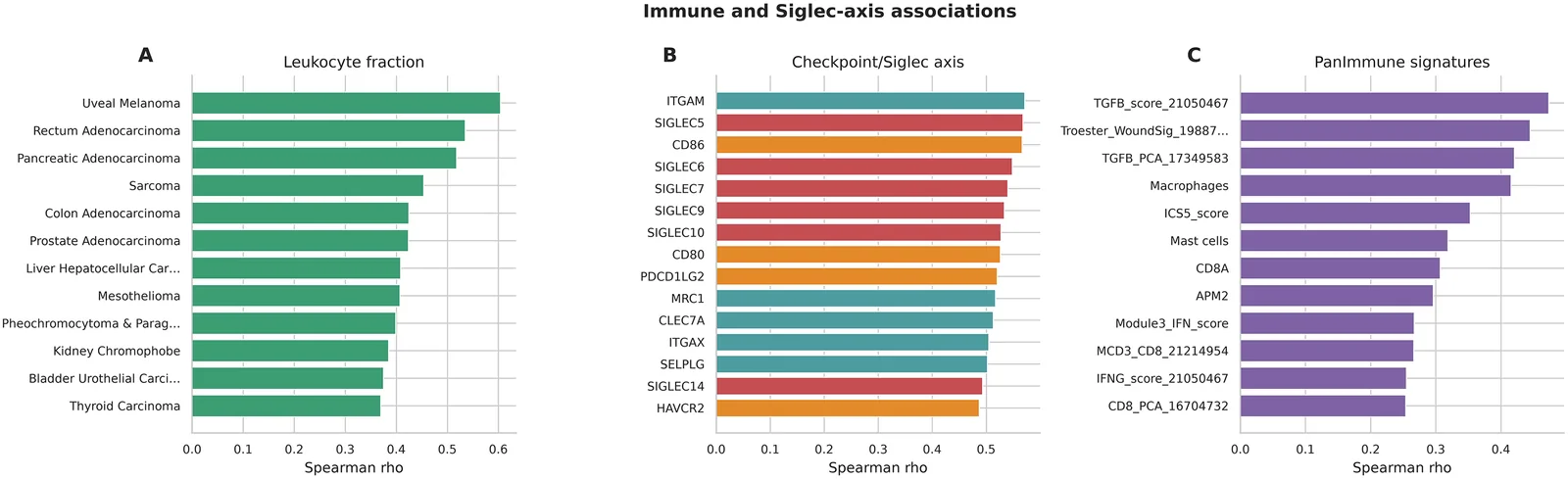
Sensitivity analyses for immune and Siglec-axis associations. **(A)** Association with leukocyte fraction. **(B)** Association with checkpoint/Siglec/myeloid-axis genes. **(C)** Association with PanImmune signatures derived from TCGA immune landscape resources. The glycoRNA-related cellular program scores are evaluated using non-overlapping sensitivity analyses.

## Discussion

The glycoRNA-related cellular program score is an expression-based composite score representing coordinated glycoRNA-related cellular programs, including RNA processing, glycan biosynthesis, trafficking, and lectin/Siglec recognition. It is designed to prioritize tumor contexts for experimental glycoRNA validation. The score serves as a context-ranking metric for glycoRNA-related cellular programs across human cancers. Two findings stand out. First, the program is preferentially elevated in selected cancer types and cellular states. Second, its most consistent association is with immune-infiltrated microenvironments enriched for Siglec and checkpoint-axis genes. These results nominate specific tumor types, cellular phenotypes, and immune pathways for targeted experimental follow-up and for direct tests of whether glycoRNA abundance or function is actually increased in these settings. The observed associations provide a transcriptional basis for prioritizing these experimental studies.

The immune-associated pattern of the glycoRNA-related cellular program was supported by complementary composition and covariate analyses (10). The positive correlations of the component modules with the total score describe coordinated transcriptional variation. Removing the lectin/Siglec component attenuated the Siglec-decoder, myeloid, and checkpoint associations, while their coefficients remained positive after adjustment for leukocyte fraction and cancer type. These observations support a cellular-program interpretation in which glycan-related, RNA-processing/trafficking, and lectin/Siglec components contribute to the observed tissue-level co-occurrence. Cancer-type-stratified results further describe variation across tumor contexts. The score therefore provides an expression-based basis for selecting settings for biochemical and spatial glycoRNA studies (12).

The GBM single-cell and spatial validation analyses add cellular-resolution and tissue-context support to the pan-cancer observations, while also highlighting that effect sizes differ by dataset and compartment. The Neftel 10X validation most strongly supports a mesenchymal/niche-state association, whereas the direct myeloid, Siglec, and checkpoint associations are small at the single-cell level and should not be overinterpreted. Elevated glycoRNA-related cellular program was detected in bulk-region and AC/MES-like malignant cell states, along with coordinated upregulation in tumor-associated macrophage compartments. These states have previously been linked to stress adaptation, pro-inflammatory signaling, and close myeloid-tumor cell crosstalk. They therefore represent reasonable candidates for direct validation, including additional patient-level pseudobulk replication, independent GBM single-cell or spatial datasets, glycoRNA enrichment or capture assays, sialic acid-dependent imaging, Siglec-Fc binding assays with RNase or sialidase sensitivity controls, and perturbation of glycan-Siglec axis components.

### Practical biochemical validation strategy

A feasible first-pass validation experiment would compare two or more high-score cancer cell lines or patient-derived GBM cultures with matched low-score controls. Cells would be metabolically labeled with an azido-sialic-acid precursor such as Ac4ManNAz, followed by click-biotin enrichment of labeled glycoconjugates and RNA extraction from the enriched fraction. Candidate glycoRNA signal can then be tested by RT-qPCR for Y RNAs or other candidate RNAs, small-RNA sequencing, or dot-blot/northern-style detection. Specificity controls should include no-label controls, RNase treatment, sialidase treatment, glycosylation inhibition or perturbation, input RNA normalization, and spike-in controls. To connect the signal to immune recognition, parallel Siglec-5/7/9-Fc binding assays should be performed with RNase and sialidase sensitivity controls, followed by macrophage adhesion, phagocytosis, or cytokine-response assays if binding is reproducible. These experiments would directly test whether high glycoRNA-related cellular program contexts contain more detectable glycoRNA-like material and whether that material contributes to Siglec-sensitive cell-surface interactions.

Several limitations are noted. First, the glycoRNA-related cellular program score summarizes glycoRNA-related cellular programs at the transcriptomic level and supports the selection of tumor contexts for quantitative biochemical glycoRNA profiling. Second, the top-100 gene-function relationships identified through literature mining are reported with evidence-confidence categories and should be interpreted within those confidence boundaries. Third, the clinical associations tested by Cox regression did not survive false discovery rate (FDR) correction and are therefore exploratory. Fourth, although independent GBM single-cell and spatial validation outputs were incorporated, the validation remains expression-based and does not directly visualize glycoRNA molecules. Higher-resolution spatial transcriptomics, matched protein/glycan imaging, or orthogonal single-cell platforms would strengthen conclusions about cellular localization. Finally, this study does not include direct perturbation experiments or biochemical confirmation of glycoRNA. The next step is experimental validation: glycoRNA enrichment or capture, multimodal imaging with tools such as sialic acid-specific probes, Siglec-5/7/9 binding assays with nuclease and glycosidase controls, and targeted perturbation of glycan biosynthesis, RNA-trafficking, or Siglec signaling components.

## Data and code availability

TCGA RNA-seq TPM data were obtained from the UCSC Xena Toil recompute resource, clinical endpoints from the TCGA Clinical Data Resource, and leukocyte fraction/PanImmune signatures from the TCGA immune landscape resource. GBM single-cell analyses used the annotated h5ad matrix described in Methods and independent GSE131928_Neftel 10X validation outputs. Spatial GBM validation was performed using the processed spatial transcriptomic samples summarized in the supplementary outputs. Processed score matrices, model outputs, plotting-ready tables, and reviewer-requested **supplementary figures** are provided as **Supplementary Tables** and **Supplementary Figures**.

## Data Availability

The datasets presented in this study can be found in online repositories. The names of the repository/repositories and accession number(s) can be found in the article/**Supplementary Material**.

## Supplementary material

Supplementary Table 1 literature metadata.csv: Table 1 literature metadata.

Supplementary Table 2 extracted entities.csv: Table 2 extracted entities.

Supplementary Table 3 extracted relations.csv: Table 3 extracted relations.

Supplementary Table 4 seed gene modules.xlsx. (Complete list of the 74 scored genes, core/support evidence categories, original modules, gene-level sources, evidence types, and TCGA coverage; accompanying sheets provide evidence definitions, source references, original-table correspondence, and the three additional seed-entry coverage records).

Supplementary Table 5 TCGA sample scores.csv: Table 5 TCGA sample scores.

Supplementary Table 6 TCGA cohort summary.csv: Table 6 TCGA cohort summary.

Supplementary Table 8 TCGA survival logrank.csv: Table 8 TCGA survival logrank.

Supplementary Table 9 TCGA multivariable cox.csv: Table 9 TCGA multivariable cox.

Supplementary Table 10 TCGA leukocyte fraction associations.csv: Table 10 TCGA leukocyte fraction associations.

Supplementary Table 11 TCGA PanImmune signature associations.csv: Table 11 TCGA PanImmune signature associations.

Supplementary Table 12 TCGA checkpoint Siglec axis correlations.csv: Table 12 TCGA checkpoint Siglec axis correlations.

Supplementary Table 13 GBM celltype enrichment.csv: Table 13 GBM celltype enrichment.

Supplementary Table 14 GBM zone enrichment.csv: Table 14 GBM zone enrichment.

Supplementary Table 15 GBM NeftelClass enrichment.csv: Table 15 GBM NeftelClass enrichment.

Supplementary Table 16 GBM single cell scores.csv.gz: GBM single-cell glycoRNA-related cellular program and module scores.

Supplementary Table 17 reviewer adjusted linear models leukocyte cancertype.csv: Adjusted non-overlapping models with leukocyte fraction and cancer-type fixed effects.

Supplementary Table 18 reviewer random effects meta analysis within cancer.csv: Within-cancer random-effects meta-analysis for immune/Siglec-free and full predictor scores.

Supplementary Table 19 reviewer nonoverlap module ablation spearman.csv: Module-ablation and non-overlapping Spearman sensitivity analyses.

Supplementary Table 20 reviewer module gene coverage.csv: Matched gene counts and module gene lists used for recomputed scoring.

Supplementary Table 21 reviewer neftel associations.csv: Independent GSE131928 Neftel 10X validation associations.

Supplementary Table 22 reviewer spatial associations.csv: Spatial GBM spot-level associations.

Supplementary Table 23 reviewer spatial neighborhood.csv: Spatial-neighborhood enrichment around high glycoRNA-related cellular program spots.

Supplementary Table 24 reviewer seed label permutation null analysis.csv: Conservative label-permutation null analysis for immune-axis outcomes.

Supplementary Table 25 Component-module correlations and leukocyte/cancer-type-adjusted associations. The workbook includes six component-to-total correlations, the 44 existing adjusted models with reproduced coefficients and 95% confidence intervals, module-removal correlations, module definitions, and analysis notes.

Supplementary Figure 1 reviewer adjusted nonoverlap betas.png: Adjusted beta estimates for immune/Siglec-free glycoRNA-related cellular program models.

Supplementary Figure 2 reviewer module ablation heatmap.png: Module-ablation sensitivity heatmap.

Supplementary Figure 3 reviewer random effects meta forest.png: Within-cancer random-effects forest plot.

Supplementary Figure 4 Component-module correlations with the glycoRNA-related cellular program score. Cells show Spearman correlations between each of the six component modules and their total composite score in 9,186 TCGA tumors from 32 cancer types. The heatmap describes score composition. Exact correlations, six-test FDR values, module definitions, and leukocyte/cancer-type-adjusted models are provided in **Supplementary Table 25**.
